# Combining angiotensin receptor blockade and enzyme replacement therapy for vascular disease in mucopolysaccharidosis type I

**DOI:** 10.1016/j.ymgmr.2023.101036

**Published:** 2023-12-14

**Authors:** Sarah C. Hurt, Moin U. Vera, Steven Q. Le, Shih-hsin Kan, Quang Bui, Patricia I. Dickson

**Affiliations:** a Washington University School of Medicine in St. Louis, MO, USA; b Lundquist Institute at Harbor-UCLA Medical Center, Torrance, CA, USA; c Southern California Permanente Medical Group, Los Angeles, CA, USA; d CHOC Research Institute, Orange, CA, USA

**Keywords:** Lysosomal storage disease, Hurler, Scheie, Hurler-Scheie, Losartan, Alpha-L-iduronidase

## Abstract

Vascular involvement in the genetic disorder mucopolysaccharidosis type I (MPS I) has features of atherosclerotic disease near branch points of arterial vasculature, such as intimal thickening with disruption of the internal elastic lamina, and proliferation of macrophages and myofibroblasts. Inflammatory pathways are implicated in the pathogenesis of vascular disease in MPS I animal models, evidenced by cytokines like CD18 and TGF-β within arterial plaques. The angiotensin II-mediated inflammatory pathway is well studied in human atherosclerotic coronary artery disease. Recent work indicates treatment with the angiotensin receptor blocker losartan may improve vascular MPS I disease in mouse models. Here, we combined losartan with the standard therapy for MPS I, enzyme replacement therapy (ERT), to measure effects on cytokines in serum and aortic vasculature. Each treatment group (losartan, ERT, and their combination) equally normalized levels of cytokines that were largely differential between normal and mutant mice. Some cytokines, notably CD30 ligand, Eotaxin-2, LIX, IL-13, IL-15, GM-CSF, MCP-5, MIG, and CCL3 showed elevations in mice treated with ERT above normal or mutant levels; these elevations were reduced or absent in mice that received losartan or combination therapy. The observations suggest that losartan may impact inflammatory cascades due to MPS I and may also blunt inflammation in combination with ERT.

## 1 Introduction

Mucopolysaccharidosis type I (MPS I) is a genetic disorder of lysosomal storage caused by deficiency of an enzyme, α-l-iduronidase, that catabolizes glycosaminoglycans in lysosomes [1]. Enzyme replacement therapy (ERT) with recombinant α-l-iduronidase and hematopoietic stem cell transplantation with healthy donor cells that can provide a source of wild-type α-l-iduronidase are available clinically to treat patients with MPS I [2]. However, these approaches do not completely eradicate disease. Residual disease burden in treated patients includes progressive disability and deformities of bones and joints that limit daily activities and cause dysfunction and pain. Cardiovascular tissues including cardiac valvular dysplasia, fibrotic cardiomyopathic changes, and arteriosclerotic-like vessel narrowing [[3], [4], [5]] also occur and respond poorly to current therapies. The cardiac and vascular manifestations continue to be a leading cause of death in treated MPS I patients [6].

Studies in animal models of several different MPS types have identified TNF-α and its transcriptional regulators as important effectors mediating inflammation in bone and joint disease [7,8]. A current model for the findings in MPS joint disease hypothesizes that accumulated glycosaminoglycans (GAG) bind to and activate toll-like receptor 4 (TLR4) leading to expression of TNF-α and matrix metalloproteinases [8,9]. Based on this model, systemic treatment with either the specific TNF-α inhibitor Remicade or the anti-inflammatory pentosane polysulfate has been shown to reduce tissue findings of joint inflammation and serum levels of TNF-α [9,10]. In parallel with the progress that has been made in understanding bone and joint disease, inflammation has also been implicated as an etiology in MPS I vascular disease [[11], [12], [13], [14]]. Aortas of MPS I mice demonstrate elastic fiber disruption, matrix metalloproteinase expression and wall weakening [[14], [15], [16]]. In the MPS I dog there are regions of intimal-medial thickening with vascular smooth muscle cells (VSMC) proliferation, internal elastic lamina disruption and extracellular matrix remodeling [11,13]. There is also evidence of increased TLR4 expression and signaling in these lesions [12,13]. These findings imply a pathophysiology similar to that found in bone and joint disease and therefore suggest the possibility of response to proven or investigational anti-inflammatory therapies [9,10].

We and others have observed increased transforming growth factor beta (TGFβ) and TLR4 expression and signaling within MPS I vascular lesions [11,12,17]. TGFβ is a known VSMC mitogen that causes proliferation of cultured VSMC and intimal hyperplasia in vivo [18]. Its presence in MPS I-related vascular lesions suggests a role for angiotensin II. The angiotensin II-mediated inflammatory pathway has been well studied in human atherosclerotic coronary artery disease and may in some cases contribute to inflammation in joints and brain as well [[19], [20], [21], [22], [23], [24], [25], [26]]. In atherosclerosis, macrophages are hypothesized to be recruited to sites of vascular endothelial damage where they secrete angiotensin II [19,22,27]. Angiotensin 1 (AT1) receptor stimulation and signaling in macrophages, endothelial cells and VSMC then leads to activation of inflammatory pathways including TLR4 and production of inflammatory cytokines including TGF-β [19,22,27,28]. AT1 receptor and TGF-β receptor signaling in VSMC both share the intermediates phosphorylated extracellular signal-regulated protein kinase 1/2 (pERK1/2) and pSMAD2 [29,30]. This suggests that blocking AT1 signaling could primarily inhibit inflammatory cytokine production and secondarily inhibit TGF-β-mediated processes such as VSMC proliferation. Angiotensin receptor blockers are a well-studied class of therapeutics that could provide an additional benefit to MPS I patients by inhibiting TGF-β signaling through pERK1/2 as they have been shown to do in Marfan syndrome [22,27,30,31]. This inflammatory mechanism therefore suggests an alternative approach for evaluating new therapies and identifying new specific biomarkers for MPS I vascular disease, such as TGF-β.

Recently, the angiotensin receptor blocker losartan was demonstrated to show a therapeutic effect in mouse models of MPS I [32,33]. Both skeletal involvement, evidenced by abnormal cranial shape, and cardiac disease, evidenced by ventricular dilation, shortening fraction, and aortic dilation, improved in treated mice. The mechanism of improvement in cardiovascular function was attributed to restoration of AKT and ERK1/2 activation [34]. However, it is unlikely that losartan could be effective monotherapy for MPS I, because it does not correct the primary defect, deficiency of the enzyme α-l-iduronidase. Here, we studied the role of angiotensin II-mediated inflammation in MPS I vascular disease and its response to treatment with losartan alone or in combination with ERT with recombinant α-l-iduronidase (laronidase) in MPS I mice.

## 2 Results

### 2.1 An exploration of change in inflammatory factors as a result of losartan administration

**Fig. 1:**
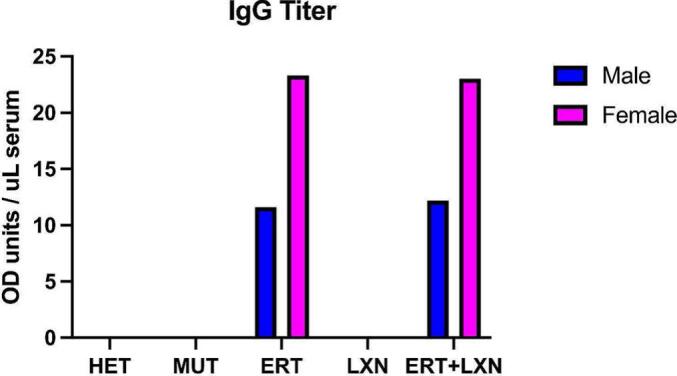
Anti-iduronidase antibodies in treated mice. Specific IgG against rhIDU in pooled serum were measured by ELISA at the 24-week timepoint (16-weeks post initial treatment).

Unaffected (*Idua*^*+/−*^) and MPS I (*Idua*^*−/−*^) mice in this study are summarized in Table 1. The ERT group received weekly 1.57 mg/kg recombinant human α-l-iduronidase (rhIDU) by tail vein The angiotensin receptor blockade losartan (LXN) group received (0.6 g/L LXN in drinking water). A separate group of MPS I mice received both treatments (ERT + LXN) at the same doses. All treatments began at eight weeks of age for a 16-week period until mice were 24 weeks of age. Serum was collected before treatment at 8 weeks of age and every 4 weeks until 24 weeks of age. Serum samples were pooled for each sex within each treatment group at 8-, 16- and 24-weeks timepoints. Mice treated with ERT developed specific antibodies against rhIDU 16 weeks post treatment (24 wks; Fig. 1) in both sexes, as previously reported.

**Table 1:** Groups of mice and their respective treatments.

Group Number	Group	Genotype	Treatment	Number of Mice	Number of Females
1	HET	+/−	Control	11	5
2	MUT	−/−	Control	10	5
3	ERT	−/−	ERT	9	3
4	LXN	−/−	Losartan	12	6
5	ERT + LXN	−/−	Losartan + ERT	11	5

**Fig. 2:**
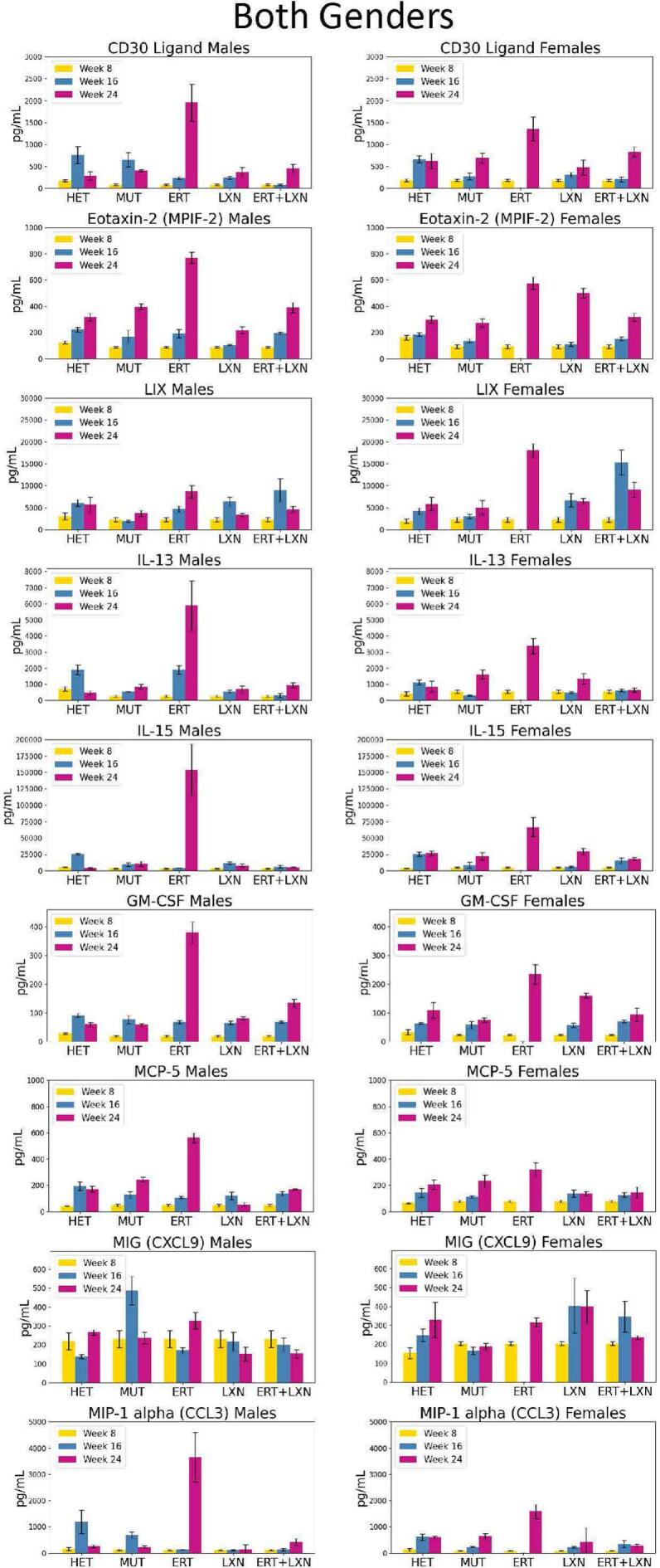
Cytokine levels are ameliorated when combining ERT and losartan. Across several cytokine measurements, there is a general trend of the ERT treated group having a higher spike in that cytokine at 24 weeks of age (16 weeks post initial treatment). The cytokine spikes in the ERT group generally are higher in males than in females. This is especially true in CD30, Eotaxin-2, IL-13, IL-15, GM-CSF, MCP-5, and MIP-1α.

**Fig. 3:**
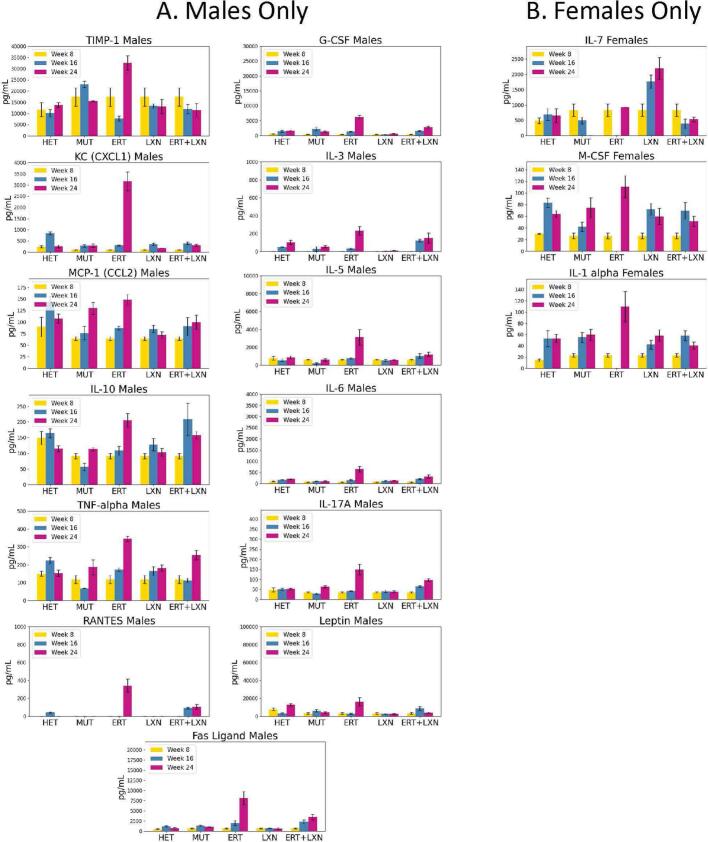
Identified improvement with combination treatment in some cytokines only in one sex. Many cytokines saw the same trend of the combination treatment leading to less inflammation in the combination treatment group at 24 weeks of age (16 weeks post treatment) only within one sex with the other sex being either an insignificant difference between the two groups or the combination group was higher in that cytokine at 24 weeks of age. (A) Cytokines where males had a significant decrease in response in the combination group when compared to the ERT alone group. (B) Cytokines where females had a significant decrease at 24 weeks of age in response in the combination treatment group when compared to the ERT alone group.

Inflammatory factors such as cytokines are an important part of the signaling network between cells and are necessary for regulating the immune system [35]. Throughout this study, we investigated impacts of cytokine levels in serum in MPS I animals administered with LXN with or without ERT treatment. Generally, a trend of elevated immune response measured in multiple cytokine levels was observed in the ERT treatment group at the 24 weeks of age (16 weeks post treatment) timepoint but not in the LXN treated group. Interestingly, when the two treatments were combined, the immune response spike generally observed in the ERT group at 24 weeks is reduced possibly due to the addition of LXN administration. We see this trend over both sexes in several cytokines including CD30, Eotaxin-2, LIX, IL-13, IL-15, GM-CSF, MCP-5, MIG, and MIP-1α (Fig. 2). This trend continued in several cytokines in one sex. In males, this was observed for KC, MCP-1, TIMP-1, TNF-α, IL-6, RANTES, and IL-10 (Fig. 3A). In females, this trend was observed for IL-7, M-CSF, and IL-1α (Fig. 3B). We also noticed that when both sexes were observed to have an immune response spike, in the ERT group, the males typically had a higher spike than females (Fig. 2). We noticed this trend in CD30, Eotaxin-2, IL-13, IL-15, GM-CSF, MCP-5, and MIP-1α. LIX had the opposite effect, in which ERT females were observed to have a higher amount of LIX than the males.

**Fig. 4:**
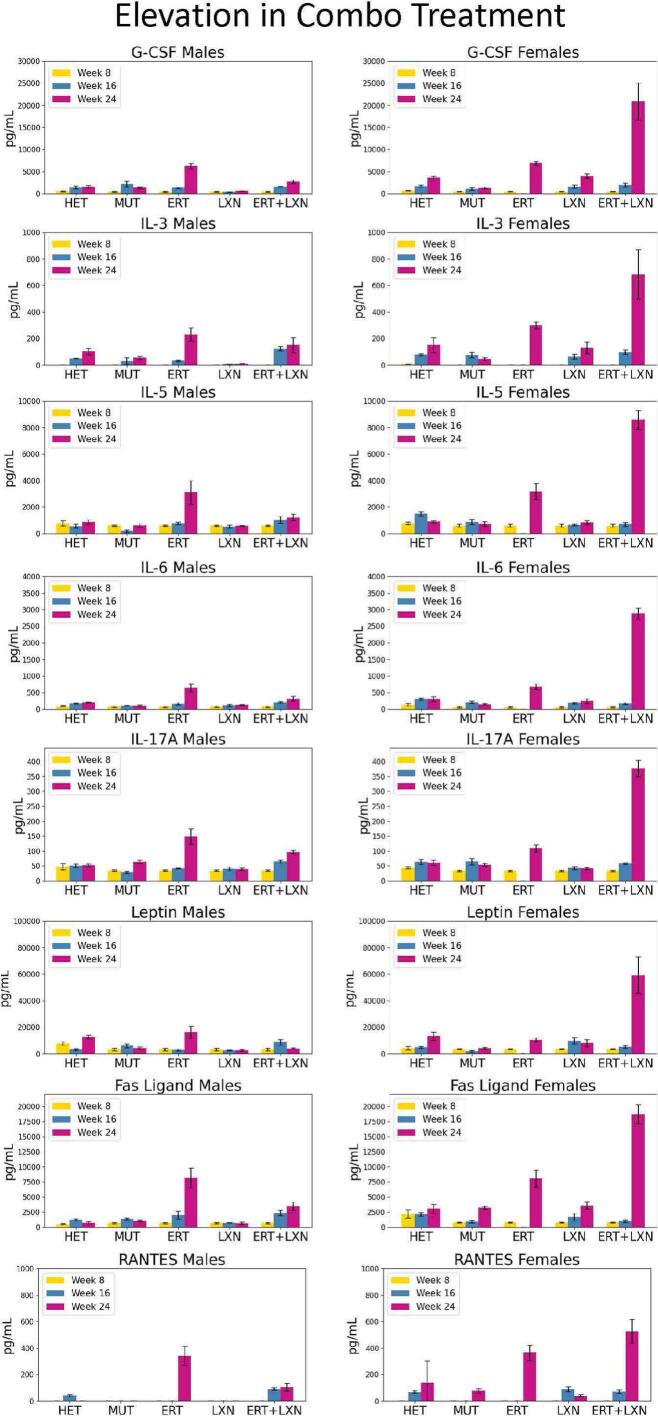
Combination treatment in females led to an increase of eight cytokines in response when compared to the ERT alone or LXN alone groups. In females, there was a trend in eight cytokines that the combination treatment group (ERT + LXN) had elevated levels of the cytokines when compared to the ERT alone group at 24 weeks of age and 16 weeks post treatment initialization. This elevation of the combination treatment was also higher than the LXN alone group as well. These cytokines were G-CSF, IL-3, IL-5, IL-6, IL-17 A, Leptin, Fas Ligand, and RANTES.

Interestingly, we also observed a significant sex bias in certain cytokines when evaluating the combination treatment. This was observed for the cytokines G-CSF, IL-3, IL-5, IL-6, IL-17, Leptin, Fas Ligand, and RANTES. All these elevation points in the combination treatment occurred in females (Fig. 4).

### 2.2 Immunohistochemistry shows reduction of TGF-β and pERK1/2 activation in all treatment groups

**Fig. 5:**
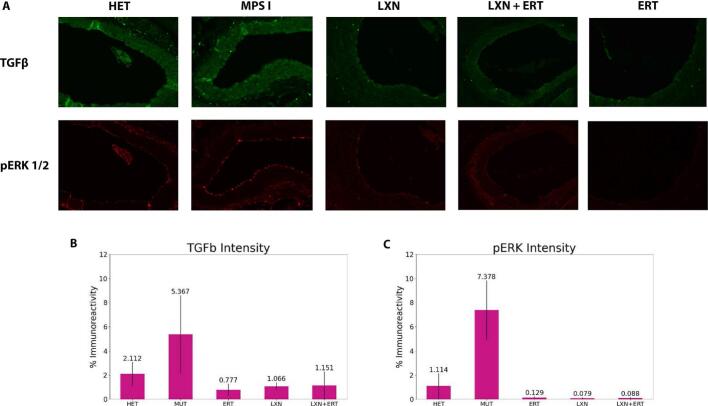
TGFβ and pERK1/2 levels are lowered with any of the treatment methods explored in this study. (A) Aortic valve sections stained for TGFβ (top row) and pERK1/2 (bottom row). All treatments showed less fluorescence than the untreated control groups (HET and MUT). (B) Graphs showing the level of intensity of TGFβ (left) and pERK1/2 (right) in each treatment group based on the immunohistochemistry. Number of mice per group evaluated via immunohistochemistry: Het – 6, MUT – 2, ERT – 6, LXN – 4, ERT + LXN – 5.

TGF-β has been implicated in MPS I related hypertrophic cardiomyopathy, and treatment with ERT alone may not be able to prevent or improve cardiomyopathic events [17]. In the present study, immunohistochemical evaluation of aortic tissue showed that untreated MUT controls had higher tissue levels of TGF-β and pERK than untreated HET mice. Levels of these markers by immunostaining were lower than MUT levels in all treatment groups, including ERT alone, with the levels of these markers in all treatment groups at or below those observed in the HET mice (Fig. 5).

## 3 Discussion

The goal of our study was to determine whether treatment with LXN might provide additive benefit over ERT alone for MPS I disease. The rationale is that inflammation triggered by MPS disease may contribute to residual disease burden. Instead, we observed elevation of several cytokines in MPS I mice treated with ERT compared to untreated MPS I (MUT) mice. Most MPS I patients receiving ERT develop anti-IDUA antibodies [36]. Fig. 1 shows that anti-IDUA antibodies developed in mice in the present study and could be contributing to an elevated immune response observed by the elevated cytokines in the ERT alone group. The combination of LXN and ERT decreased inflammatory response in several cytokines that were noted to be elevated in mice treated with ERT alone (Fig. 2). This was observed across sexes for several cytokines that have not previously been associated with angiotensin II-related inflammation.

Angiotensin II is known to regulate the synthesis of proinflammatory cytokines and chemokines including TNF-α, IL-6, RANTES, and MCP-1 [37]. An angiotensin receptor blocker, such as LXN, is thought to lessen inflammation from these cytokines [38]. In our study, we observed a reduction in these inflammatory factors only in males aged 24 weeks, which corresponded to 16 weeks after initial treatment with ERT + LXN, when compared to mice only treated with ERT. MCP-1 and TNF-α, which have been previously reported as elevated cytokines when in the presence of angiotensin II-induced inflammation, are included in the lowered trend in males in our study indicating that the treatment may be leading to less inflammation from angiotensin II related pathways [39]. However, IL-6 and RANTES in female mice were elevated in mice treated with ERT + LXN compared to ERT alone. This observation could suggest a sex-based difference. MPS I mice have been reported to show sex differences in phenotype, including increased rate of aortic insufficiency and matrix metalloproteinase-12 content in aortas of male mice [40]. There are sex differences in human immune responses [41]. It is also possible that one or more outliers in the pooled sample or other variability contributed to this result.

Our study has several limitations. Serum samples were pooled for cytokine analysis due to limited amount of serum available for each timepoint. This design limits our ability to determine statistical significance and precludes the detection of potential outliers in our samples. In addition, we were only able to perform pathological assessments of immune mediators, TGF-β and pERK1/2, in sections of the aortic valves due to the large vascular structure available for staining in the aortic valve for these markers.

There are several studies indicating the anti-inflammatory effects of losartan [[42], [43], [44]]. Additionally, there are preclinical studies showing losartan alone can improve ventricular contraction and and cardiac function [32,33]. Osborn et al. 2017 also demonstrates a sex difference between cardiac function in untreated knockout mice [32]. They found that perturbations in the renin angiotensin system were more prominent in males and losartan led to more critically improved cardiac function in males than in females. Critically, unlike our study, neither of these studies explored the combination of losartan with ERT and how that may affect cytokine production. Importantly, it is worth to note that both ERT for MPS I and losartan are FDA approved treatments [45], and a potential clinical application of combining these treatments together could be studied for patients with MPS I. Future work could study whether combining the two treatments would allow for amelioration of an immune response that might occur as a result of ERT, and whether this combination would result in additional therapeutic benefit over ERT alone.

## 4 Material and methods

The MPS I mouse (B6.129-*Idua*^*tm1Clk*^/J) was maintained on a C57BL/6 background. *Idua*^*+/−*^ females were crossed with *Idua*^*−/−*^ males to obtain homozygous affected (*Idua*^*−/−*^) mice and heterozygous (*Idua*^*+/−*^) as unaffected controls. Genotype was determined using the following primers: oIMR1451: 5′-GGAACTTTGAGACTTGGAATGAACCAG-3′, oIMR1452: 5′-CATTGTAAATAGGGGTATCCTTGAACTC-3′, and oIMR1453: 5′-GGATTGGGAAGACAATAGCAGGCATGCT-3′. MPS I mice received weekly recombinant human α-l-iduronidase (rhIDU) ERT at a dose of 1.57 mg/kg body weight from week 8 to 24 weeks of age via tail vein injection. Controls received weekly saline injections. To prevent hypersensitivity reactions, 5 mg/kg diphenhydramine via intraperitoneal injection was given prior tail vein injection. Mice in losartan groups received 0.6 g/L of the angiotensin receptor blockade losartan(Toronto Research Chemicals, Inc. Cat# L470500) in drinking water.

Blood samples were collected through the facial submandibular vein every 4 weeks beginning at week 8 (prior to any treatment). Serum samples from weeks 8, 16, and 24 of each group were pooled to generate five samples per timepoint (grouped by genotype, sex, and treatment). Measurement of anti-iduronidase IgG antibodies was conducted by ELISA on week 24 serum samples as previously described [46]. A mouse inflammation array (RayBiotech, Cat# QAM-INF-1-1) was used to analyze 40 different inflammatory markers on serum samples from weeks 8, 16, and 24 according to the manufacturer's instructions. Pre-treatment serum samples from MPS I animals were pooled and unaffected carriers controls (week 8) were pooled as separate groups. Slides were scanned with a gene microarray laser scanner and densitometric data were analyzed with Q-Analyzer® software (RayBiotech Due to space limitations on the array, ERT females at week 16 was not included in the study and were zeroed out in the graphics. Additionally, cytokine data below the limit of detection (LOD) were also zeroed out in the graphics and standard deviation in the study represents the multiple scanning points generated for each cytokine.

Immunohistochemistry was performed on 6-μm-thick circular sections of the aorta and blood vessels cut transversally through paraffin blocks, with antibody against TGF-β (21898–1-AP, ThermoFisher Scientific; 1:50 dilution) and pERK1/2 (14–9109-82; ThermoFisher Scientific; 1:50 dilution) followed by appropriate secondary antibodies (goat anti-rabbit Alexa Fluor-488, #A11008; and goat anti-mouse 546 #A11003; 1:200 dilution, ThermoFisher Scientific). Slides were mounted with DAPI Fluoromount-G (SouthernBiotech) after applying with autofluorescence quencher TrueBlack (Biotium) Slides were scanned using a Zeiss Axio Scan.Z1 (Zeiss, Jena, Germany) at 10× magnificationand scanned imaged was processed for individual antibodies with ImagePro Premier 10 software (Media Cybernetics, Chicago, IL, USA).The thresholds settings were then applied uniformly across all images and the results were reported as percentage positive immunoreactivity in blood vessels [47].

## Study approval

All animal experiments were approved by the institutional animal use committee at the Los Angeles Biomedical Research Institute at Harbor-UCLA Medical Center (now the Lundquist Institute) in Torrance, California.

## Credit authorship contribution statement

**Sarah C. Hurt:** Formal analysis, Investigation, Writing – original draft, Writing – review & editing. **Moin U. Vera:** Conceptualization, Funding acquisition, Methodology, Writing – review & editing. **Steven Q. Le:** Investigation, Methodology, Writing – review & editing. **Shih-hsin Kan:** Investigation, Methodology, Writing – review & editing. **Quang Bui:** Investigation, Methodology. **Patricia I. Dickson:** Conceptualization, Formal analysis, Funding acquisition, Methodology, Project administration, Resources, Supervision, Writing – review & editing.

## Declaration of Competing Interest

This study was supported by a grant from Sanofi Corporation. Dr. Dickson is a listed inventor on Patent #USSN 15/946,505; 0WVR-223,143-US for enzyme replacement therapy for mucopolysaccharidosis IIID. Dr. Dickson also receives research support from M6P Therapeutics, Alnylam, and Mandos Health.

## Data Availability

Data will be made available on request.
